# Non-pharmacological Interventions for Pain-Related Anxiety and Distress in Emergency Department Patients: A Systematic Review With Narrative Synthesis

**DOI:** 10.7759/cureus.112774

**Published:** 2026-07-16

**Authors:** Afnan M Hamed, Reem E Elshaikh, Amer A Laradhi, Ahmad M Fnjan, Anas M Berro, Asim Ahmed, Rayan Saeed

**Affiliations:** 1 General Practice, Hail General Hospital, Hail, SAU; 2 Emergency Medicine, Johns Hopkins Aramco Healthcare, Al-Hasa, SAU; 3 Internal Medicine, King Abdulaziz University Hospital, Jeddah, SAU; 4 General Practice, King Abdulaziz University Hospital, Jeddah, SAU; 5 Faculty of Medicine, Beirut Arab University, Beirut, LBN; 6 Medicine and Surgery, University of Gezira, Wad Madani, SDN; 7 General Medicine, The National Ribat University, Khartoum, SDN

**Keywords:** acute pain, anxiety, emergency department, music therapy, non-pharmacological interventions

## Abstract

Pain-related anxiety, fear, and distress are common challenges in emergency departments and may influence pain perception, patient cooperation, satisfaction, and the overall care experience. This systematic review and narrative synthesis examined evidence on non-pharmacological interventions for pain intensity, emotional distress, patient experience, and analgesic-related outcomes among emergency department patients. A structured search was conducted from database inception to May 2026 using PubMed/MEDLINE, Semantic Scholar, Google Scholar, Elicit, publisher webpages, DOI checking, and citation tracking. Only English-language reports with sufficient extractable data were included. Eligible studies included original interventional, feasibility, pilot, quasi-experimental, quality improvement, and qualitative studies conducted in emergency department settings. Twenty-five studies were included in the qualitative synthesis, including randomized, pilot randomized, non-randomized, quality improvement, feasibility, and qualitative evidence. Interventions were grouped into music and sound-based interventions, virtual reality and distraction-based interventions, and acupuncture-based interventions, reflecting the main non-pharmacological approaches identified in the included emergency department literature. Overall, findings were more consistently favorable for short-term anxiety, fear, distress, coping, comfort, satisfaction, and patient experience than for pain intensity, which remained variable across studies. No pooled effect estimate was calculated because of substantial clinical and methodological heterogeneity, and individual study findings varied by intervention type, comparator, population, setting, outcome measure, and timing of assessment. Music and sound-based interventions showed possible short-term benefits for anxiety, comfort, and patient experience, but pain findings were mixed. Virtual reality and distraction-based interventions showed the most consistent favorable findings in pediatric and procedural contexts, particularly for anxiety, fear, coping, and distress, although analgesic effects were heterogeneous. Acupuncture showed preliminary favorable findings mainly in selected adult acute pain settings, but the evidence was limited by feasibility, quality improvement, and qualitative designs. Risk-of-bias concerns included small samples, limited blinding, subjective outcomes, heterogeneity in measurement tools, and short follow-up periods. Because GRADE was not performed, certainty of evidence was interpreted narratively and should be considered limited. Current evidence suggests possible short-term benefits from non-pharmacological interventions for pain-related emotional distress and patient experience in selected emergency department contexts, but definitive analgesic effectiveness, sustained benefit, and analgesic-sparing effects remain uncertain. Further adequately powered pragmatic trials are needed to determine clinically meaningful effects, analgesic-use and opioid-sparing outcomes, and real-world implementation feasibility.

## Introduction and background

Pain in the emergency department is not only a symptom requiring a rapid clinical response but also a contributor to distress, dissatisfaction, cooperation difficulties, and perceived quality of care. A large adult emergency department study reported that more than half of patients presented with pain at triage, emphasizing that acute pain is a routine and high-volume emergency care problem rather than a marginal complaint [1]. In this setting, pain perception is shaped by clinical contributors such as injury severity and procedural intensity, emotional contributors such as anxiety, fear, previous pain experience, and perceived loss of control, and care-process contributors such as waiting time, communication, and reassurance [2]. This problem is particularly important in children, older adults, and patients undergoing invasive procedures, in whom pain and distress can be difficult to assess and may be intensified by unfamiliar surroundings, procedural fear, and separation from usual support systems [3].

Emergency care has traditionally relied on pharmacological analgesia, including acetaminophen, non-steroidal anti-inflammatory drugs, local anesthetics, and opioids when clinically indicated. However, contemporary emergency medicine increasingly recognizes that pharmacological treatment alone may not fully address the emotional, cognitive, and experiential dimensions of acute pain [4]. This distinction is important because analgesic medications primarily target nociceptive or inflammatory pathways, while fear, uncertainty, attentional focus, autonomic arousal, and perceived loss of control may continue to amplify the pain experience. This is especially relevant in the context of opioid stewardship, where clinicians are encouraged to relieve pain effectively while avoiding unnecessary opioid exposure and its associated risks. At the same time, patient-centered research indicates that many emergency department patients are willing to try non-pharmacological approaches for pain relief, particularly when these interventions are recommended by healthcare providers and presented as supportive options within usual care [5].

Non-pharmacological interventions may support emergency pain care by addressing aspects of the pain experience that conventional analgesics do not fully target. These interventions may reduce attentional focus on pain, lower anxiety and autonomic arousal, improve emotional regulation, enhance perceived control, support coping, and make painful procedures more tolerable. They are therefore best framed as approaches that may support clinically indicated analgesic care rather than as substitutes for necessary pharmacological treatment. A previous systematic review and meta-analysis of adult emergency department studies suggested that non-pharmacological interventions may reduce pain, although the evidence was heterogeneous and varied by intervention type and study quality [6]. Pediatric emergency literature has also highlighted the value of distraction and comfort-oriented approaches for painful procedures because children’s pain behaviors, fear, and cooperation are influenced by developmental stage, caregiver presence, and the clinical environment [7].

Among specific modalities, music-based interventions have received growing attention in emergency care because they may be relatively low-burden and adaptable in some settings. However, music interventions are not uniform, and effects may differ according to whether music is self-selected, prerecorded, therapist delivered, delivered through a smartphone application, or used as a structured sound-based intervention. Acceptability may also vary by patient age, illness severity, cultural preference, noise level, and emergency department workflow. A systematic review of music interventions in pediatric and adult emergency departments suggested that music may reduce pain and anxiety, although pooled effects were limited by heterogeneity in populations, procedures, intervention delivery, and outcome tools [8].

Virtual reality has also emerged as a non-pharmacological approach for procedural pain and anxiety in emergency and acute-care settings because immersive environments may provide stronger attentional engagement than more passive distraction approaches. Unlike music or simple distraction, virtual reality may create a more absorbing sensory experience that redirects attention away from the procedure and may improve coping during painful or anxiety-provoking care. An emergency department-focused systematic review and meta-analysis found that virtual reality interventions were associated with reduced pain scores, but it also emphasized variability between adult and pediatric studies and the need for stronger trials [9]. A broader systematic review and meta-analysis of virtual reality for acute procedural pain similarly suggested potential benefit, while highlighting the importance of clinical context, comparator choice, and outcome measurement [10].

Beyond music and virtual reality, emergency departments have also explored acupuncture, relaxation, breathing strategies, communication-based support, guided imagery, and other behavioral or sensory approaches. However, transferability to routine emergency department practice is not automatic because emergency care is time-sensitive, crowded, unpredictable, and often constrained by staffing and workflow pressures. Emergency medicine guidance continues to emphasize early pain recognition, reassessment, and multimodal management, creating a practical rationale for evaluating non-pharmacological approaches that may support, but should not delay, standard analgesic care [11].

Despite this growing interest, important gaps remain. Previous reviews have often focused on single modalities, such as music or virtual reality, specific patient groups, such as pediatric procedural pain, or broad pain intensity outcomes [6-10]. Fewer reviews have integrated the main non-pharmacological intervention categories studied in emergency department settings while also examining pain-related emotional distress, patient experience, feasibility, and analgesic-related outcomes together. The novelty of this review is that it synthesizes evidence across music and sound-based interventions, virtual reality and distraction-based interventions, and acupuncture-based interventions while distinguishing pain intensity from related but separate outcomes such as anxiety, fear, distress, coping, comfort, satisfaction, patient experience, and analgesic use. This distinction is important because these outcomes may respond differently to non-pharmacological interventions and may carry different levels of certainty. Panic-like distress is also rarely measured separately, even though acute panic-like symptoms can occur in emergency settings. Adult non-procedural pain, older adults, caregiver outcomes, and implementation feasibility also remain less consistently synthesized. Therefore, a focused systematic review with narrative synthesis is needed to bring together the evidence on the main non-pharmacological interventions used for pain-related anxiety and distress in emergency department patients across different intervention types and patient groups.

This systematic review aimed to synthesize evidence on non-pharmacological interventions for pain intensity and pain-related emotional distress among emergency department patients. The main intervention categories of interest were music and sound-based interventions, virtual reality and distraction-based interventions, and acupuncture-based interventions, reflecting the principal approaches identified in the emergency department literature. Secondary outcome domains included coping, comfort, satisfaction, patient experience, analgesic-related outcomes, and differences by intervention type, patient group, emergency department setting, and outcome measurement tool.

## Review

Methods

This systematic review was conducted in accordance with the Preferred Reporting Items for Systematic Reviews and Meta-Analyses (PRISMA) 2020 statement [12]. A completed PRISMA 2020 checklist was prepared, and detailed domain-level risk-of-bias assessments are presented in the Results section with the corresponding risk-of-bias tables. The review question, eligibility criteria, search strategy, screening process, data extraction plan, synthesis approach, and risk-of-bias assessment strategy were defined before final data extraction. This review was not prospectively registered in PROSPERO, OSF, or another registry; therefore, it was reported as an unregistered systematic review. However, the eligibility criteria, screening process, data extraction approach, synthesis method, and risk-of-bias assessment procedures are reported transparently. The final search was conducted on May 16, 2026.

Research Question and PICO Framework

The review question was: Among emergency department patients with acute pain, procedural pain, or pain-related emotional distress, what evidence is available on non-pharmacological interventions compared with usual care, standard analgesia alone, no intervention, or alternative non-pharmacological approaches for pain intensity, pain-related emotional distress, patient experience, and analgesic-related outcomes?

The PICO framework used to define eligibility is summarized in Table 1.

**Table 1:** PICO framework for study eligibility PICO, Population, Intervention, Comparator, and Outcomes.

PICO element	Definition used in this review
Population	Patients treated in an emergency department, emergency room, emergency ward, pediatric emergency department, geriatric emergency department, or emergency observation unit with acute pain, procedural pain, or pain-related emotional distress, including anxiety, fear, distress, or panic-like distress when reported
Intervention	Non-pharmacological interventions used in emergency department settings to address pain intensity, pain-related emotional distress, coping, comfort, satisfaction, patient experience, or analgesic-related outcomes. Broader non-pharmacological approaches were eligible if they met the review criteria, but the final synthesis was organized around the main intervention categories identified in the included studies: music and sound-based interventions, virtual reality and distraction-based interventions, and acupuncture-based interventions
Comparator	Usual care, standard analgesia alone, no intervention, noise cancellation, passive control, standard distraction, child life support, another non-pharmacological intervention, or pre-intervention baseline in single-arm studies
Outcomes	Primary outcome domains were pain intensity and pain-related emotional distress, including anxiety, fear, and distress when reported. Secondary outcome domains included coping, comfort, satisfaction, perceived comfort, patient or caregiver experience, service quality, analgesic requirement, additional local anesthetic use, adverse effects, feasibility, acceptability, implementation outcomes, and panic-like distress when specifically reported
Study designs	Randomized controlled trials, pilot randomized trials, quasi-experimental studies, prospective cohort studies, before-after studies, feasibility studies, service evaluations, quality improvement studies, and qualitative studies reporting original outcome data

Eligibility Criteria

Studies were eligible if they included emergency department patients with acute pain, procedural pain, or pain-related anxiety, fear, or distress who received a non-pharmacological intervention. Relevant outcome domains included pain intensity, pain-related emotional distress, coping, comfort, satisfaction, patient or caregiver experience, analgesic-related outcomes, adverse effects, feasibility, acceptability, and implementation-related outcomes when reported. Studies were included when the emergency department population was clearly identifiable or when emergency department data were reported separately.

Studies were excluded if they were conducted outside emergency department settings unless emergency department participants or emergency department outcome data were reported separately. Studies were also excluded if they focused only on chronic psychiatric disorders without acute pain-related anxiety or distress, evaluated pharmacological-only pain management without a non-pharmacological component, or were reviews, editorials, commentaries, letters, case reports, protocols without completed results, conference abstracts without completed full-text reports, or records without sufficient extractable outcome data. Only completed full-text English-language reports with sufficient extractable information were included in the final synthesis. Studies were not excluded on the basis of methodological quality or risk-of-bias assessment. Risk-of-bias assessment was performed after study inclusion and was used to inform interpretation of the evidence rather than to determine eligibility.

Information Sources and Search Strategy

A structured search was performed using PubMed/MEDLINE, Semantic Scholar, Google Scholar, Elicit, DOI and publisher webpages, and supplementary citation tracking. Searches were conducted from database inception to May 16, 2026, with no publication date restriction. Only English-language full-text reports with sufficient extractable outcome data were included. The PubMed/MEDLINE search was primarily keyword-based and combined emergency department terms, pain-related terms, anxiety, fear, distress terms, and intervention-specific keywords. The search was designed to identify original studies evaluating non-pharmacological interventions in emergency department settings.

Google Scholar was used as a supplementary search source. For each relevant search string, the first 200 Google Scholar results were screened using default relevance-based sorting, or screening was stopped earlier when the retrieved records became clearly unrelated to the review question. Date-based sorting was not used as the main screening order. Elicit was used only as a supplementary discovery tool to identify potentially relevant studies and was not treated as a bibliographic database. Any potentially eligible records identified through Elicit were verified using DOI records, publisher webpages, citation details, or full-text reports when available. Supplementary searching also included citation tracking of included studies, reference-list screening of relevant reviews, DOI checking, and publisher-page verification.

The search strategy is summarized in Table 2.

**Table 2:** Search strings used to identify eligible studies Searches were conducted from database inception to May 16, 2026, with no publication date restriction. Only English-language full-text reports with sufficient extractable outcome data were included. Google Scholar results were screened using default relevance-based sorting, and the first 200 results for each relevant search string were reviewed, or screening was stopped earlier when records became clearly unrelated to the review question. Elicit was used only as a supplementary discovery tool and was not treated as a bibliographic database. DOI, digital object identifier; VR, virtual reality.

Search source	Search string or search approach
PubMed/MEDLINE	("Emergency Service, Hospital" OR "emergency department" OR "emergency room" OR "emergency ward" OR "accident and emergency") AND ("acute pain" OR "procedural pain" OR pain OR anxiety OR fear OR distress OR panic) AND ("non-pharmacological" OR "nonpharmacological" OR "music therapy" OR music OR "virtual reality" OR distraction OR relaxation OR breathing OR "deep breathing" OR acupuncture OR "therapeutic communication" OR "guided imagery" OR hypnosis)
Google Scholar and Semantic Scholar	("emergency department" OR "emergency room" OR "emergency ward" OR "accident and emergency") AND ("acute pain" OR "procedural pain" OR pain OR "pain-related anxiety" OR anxiety OR fear OR distress OR panic) AND ("non-pharmacological" OR "nonpharmacological" OR "music therapy" OR music OR "virtual reality" OR distraction OR relaxation OR "breathing technique" OR "deep breathing" OR acupuncture OR "therapeutic communication" OR "guided imagery" OR hypnosis)
Elicit	Elicit was used as a supplementary discovery tool to identify potentially relevant studies using combinations of emergency department, pain, anxiety, distress, and non-pharmacological intervention terms. Potentially eligible records identified through Elicit were verified using DOI records, publisher webpages, citation details, or full-text reports when available.
Music-focused search	("emergency department" OR "emergency room" OR "emergency ward") AND ("music therapy" OR music OR "music intervention" OR "sound composition") AND (pain OR anxiety OR stress OR distress OR satisfaction)
Virtual reality-focused search	("emergency department" OR "pediatric emergency department" OR "emergency room") AND ("virtual reality" OR VR OR distraction) AND (pain OR anxiety OR fear OR distress OR coping OR venipuncture OR cannulation OR "needle procedure")
Acupuncture-focused search	("emergency department" OR "emergency room") AND acupuncture AND ("acute pain" OR pain OR anxiety OR stress OR distress OR "musculoskeletal pain")
Supplementary searching	Citation tracking of included studies, reference-list screening of relevant systematic reviews, DOI checking, and publisher-page verification were used to identify and verify additional potentially eligible studies.

Study Selection

All retrieved records were screened in two stages. First, titles and abstracts were screened against the eligibility criteria. Second, potentially eligible studies were assessed using full-text reports, DOI records, publisher pages, and citation details. Duplicate records were removed manually using a spreadsheet-based screening file. Duplicate checking was based on title, author names, year of publication, DOI, and publisher record where available.

Two reviewers independently screened and selected studies. Disagreements were resolved through discussion and consensus, and no third-reviewer adjudication was required. A formal inter-reviewer agreement statistic, such as Cohen’s kappa, was not calculated. Where duplicate or overlapping reports were identified, the most complete peer-reviewed full-text report was retained.

Data Extraction

Two reviewers independently extracted data from each included study using a standardized spreadsheet-based extraction form. The extraction form was piloted on a small number of included studies before final extraction, and minor adjustments were made to ensure that all relevant variables were captured consistently. Extracted variables included author and year, country, emergency department setting, study design, sample size, population characteristics, intervention type, comparator, outcome domains, measurement tools, main findings, adverse effects, feasibility outcomes, patient or caregiver satisfaction, analgesic-related outcomes, and risk-of-bias judgment.

Extracted data were reviewed for completeness and consistency by both reviewers, and disagreements were resolved through discussion and consensus. Study authors were not contacted for missing data. Only completed full-text reports with sufficient extractable information were included. Where full-text reports provided narrative findings without extractable summary statistics, the direction of effect and statistical significance were recorded cautiously.

The main intervention categories were music and sound-based interventions, virtual reality and distraction-based interventions, and acupuncture-based interventions. Outcomes were grouped into pain intensity, pain-related emotional distress, coping, comfort, satisfaction, perceived comfort, patient or caregiver experience, service quality, analgesic use, adverse effects, feasibility, acceptability, and implementation-related outcomes. When available, numerical outcome data such as mean scores, standard deviations, p-values, effect sizes, and confidence intervals were extracted.

Risk-of-Bias Assessment

Risk of bias was assessed according to study design using established appraisal tools [13-15]. Randomized controlled trials and pilot randomized trials were assessed using the Cochrane Risk of Bias 2 tool [13]. Non-randomized prospective studies, quasi-experimental studies, before-after studies, feasibility studies, service evaluations, and quality improvement studies were assessed using Risk Of Bias In Non-randomized Studies of Interventions (ROBINS-I) [14]. The qualitative study was assessed using the Critical Appraisal Skills Programme (CASP) qualitative checklist [15].

Each study was assessed across domains relevant to its design, including randomization process, deviations from intended interventions, missing outcome data, measurement of outcomes, selection of reported results, confounding, selection bias, classification of interventions, and bias due to lack of blinding. RoB 2 judgments were categorized as low risk of bias, some concerns, or high risk of bias. ROBINS-I judgments were categorized as low, moderate, serious, critical, or no information. CASP qualitative appraisal findings were summarized narratively because CASP does not assign a formal overall risk-of-bias category. Detailed domain-level risk-of-bias assessments were prepared for each applicable tool, while summary judgments are presented in the Results section.

Data Synthesis

A narrative synthesis was performed because the included studies differed substantially in population, intervention type, comparator, clinical setting, outcome instruments, measurement timing, and study design. Direct meta-analysis was not performed because of substantial clinical and methodological heterogeneity across studies. Findings were synthesized by intervention type: music and sound-based interventions, virtual reality and distraction-based interventions, and acupuncture-based interventions. Additional synthesis considered patient age group, clinical context, procedural versus non-procedural pain, outcome type, feasibility, safety, acceptability, patient experience, and analgesic-related outcomes.

Because the review included randomized trials, pilot trials, non-randomized studies, feasibility studies, service evaluations, quality improvement studies, and qualitative evidence, study designs were not treated as equivalent sources of causal evidence. Randomized and controlled studies were used primarily to interpret intervention effectiveness, while feasibility studies, service evaluations, quality improvement studies, and qualitative studies were used mainly to contextualize feasibility, acceptability, implementation, and patient or caregiver experience. Causal effectiveness conclusions were interpreted primarily from randomized or controlled studies.

Certainty of Evidence

A formal GRADE assessment was not performed because the included evidence comprised mixed study designs, heterogeneous interventions, different comparators, variable outcome measures, and different assessment timings. Certainty of evidence was therefore interpreted narratively based on study design, consistency of findings, risk of bias, sample size, directness of evidence, and outcome measurement heterogeneity. This issue was addressed in the Discussion and Limitations sections, and conclusions were worded cautiously to reflect the limited certainty of evidence.

Ethical Considerations

Ethics approval was not required because this systematic review used previously published data. No individual patient-level data were collected.

Results

Study Selection

**Figure 1:**
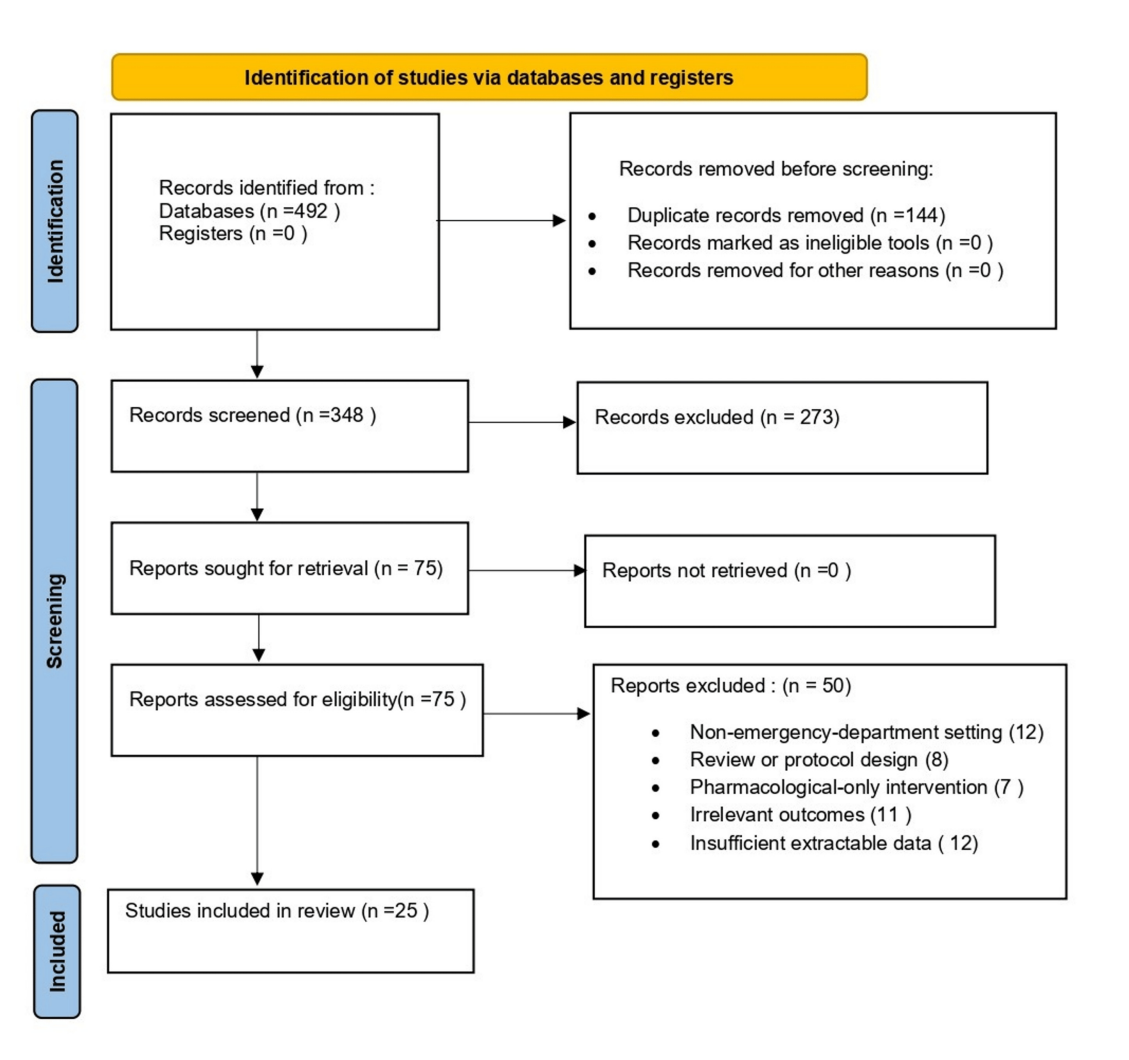
PRISMA 2020 flow diagram of study selection PRISMA, Preferred Reporting Items for Systematic Reviews and Meta-Analyses; ED, emergency department.

A total of 492 records were identified through database searching, supplementary searching, and citation tracking. After removal of 144 duplicate or clearly ineligible records, 348 records were screened by title and abstract. Of these, 273 records were excluded because they did not meet the eligibility criteria. Seventy-five full-text articles were assessed for eligibility, and 50 were excluded for the following reasons: non-emergency department setting (n = 12), review or protocol (n = 8), pharmacological-only intervention (n = 7), irrelevant outcomes (n = 11), and insufficient extractable data (n = 12). Finally, 25 original studies were included in the qualitative synthesis [16-40]. The study selection process is summarized in Figure 1.

Study Characteristics

The 25 included studies evaluated non-pharmacological interventions delivered in adult, pediatric, geriatric, and mixed emergency department settings. The evidence base included randomized controlled trials, pilot randomized trials, quasi-experimental studies, prospective cohort or feasibility studies, quality improvement data, service evaluation, and qualitative evidence. Because these designs answer different methodological questions, they were not treated as equivalent sources of effectiveness evidence. Randomized and controlled studies were interpreted primarily for intervention effects, while feasibility, quality improvement, service evaluation, and qualitative studies were used mainly to contextualize feasibility, acceptability, implementation, and patient or caregiver experience. Interventions were grouped into music and sound-based interventions, virtual reality and distraction-based interventions, and acupuncture-based interventions. The main characteristics of the included studies are summarized in Table 3.

**Table 3:** Characteristics and data extraction summary of included studies CAMPIS-SF, Child-Adult Medical Procedure Interaction Scale-Short Form; CASP, Critical Appraisal Skills Programme; CHEOPS, Children’s Hospital of Eastern Ontario Pain Scale; ED, emergency department; FLACC, Face, Legs, Activity, Cry, Consolability scale; IV, intravenous; NRS, numerical rating scale; RoB 2, Cochrane Risk of Bias 2 tool; ROBINS-I, Risk Of Bias In Non-randomized Studies of Interventions; STAI, State-Trait Anxiety Inventory; VAS, visual analogue scale; VR, virtual reality.

Study	Country/setting	Design	Population/sample	Intervention	Comparator	Main outcomes/tools	Key findings	Risk-of-bias tool
Angkoontassaneeyarat et al., 2025 [16]	Thailand, ED	Randomized controlled trial	ED patients with pain complaints; n = 63	Music therapy plus standard analgesia	Standard analgesia without music therapy	Pain, anxiety, satisfaction, ED service quality	Music therapy reduced pain and anxiety and improved perceived ED service quality	RoB 2
Goldfine et al., 2025 [17]	USA, ED	Pilot randomized trial	Adults with acute musculoskeletal back pain; n = 40	Self-selected music	Noise cancellation	Pain and anxiety numerical scores	Music reduced post-intervention anxiety and pain compared with control	RoB 2
Chai et al., 2020 [18]	USA, ED observation unit	Prospective study	ED observation-unit patients with pain; n = 81	Smartphone-based music app	Supervised versus unsupervised use	Pain, anxiety, acceptability	Music app produced modest reductions in pain and anxiety and was acceptable	ROBINS-I
Hedayati et al., 2023 [19]	Iran, emergency wards	Randomized controlled trial	Adults undergoing hand or foot suturing; n = 60	Traditional Iranian wordless music	Usual suturing care	VAS pain, anxiety scores	Anxiety was lower with music; pain reduction was not statistically significant	RoB 2
Bakhshandeh et al., 2024 [20]	Iran, ED	Randomized controlled trial	Children aged 3 to 6 undergoing wound suturing; n = 60	Music therapy	Standard care	CHEOPS pain, parental STAI anxiety	Children’s pain and parental anxiety decreased clinically in the music group, although between-group differences were not statistically significant	RoB 2
Mandel et al., 2019 [21]	USA, hospital ED	Service evaluation/matched comparison	ED patients receiving music therapy; >1,500 service encounters, with survey subgroup	Therapist-delivered music therapy	Matched non-music comparison	Patient satisfaction, stress, pain, staff perceptions	Stress and pain improved among music therapy patients; formal satisfaction differences were not statistically significant	ROBINS-I
Ettenberger et al., 2025 [22]	Colombia, ED waiting areas	Multicenter randomized clinical trial	Adult patients and caregivers; n = 430 total	Live environmental music therapy or prerecorded music	Standard care	STAI-6, VAS stress, VAS pain, well-being scales	Both music interventions reduced anxiety, stress, and pain; live music showed broader well-being benefits	RoB 2
Birrenbach et al., 2022 [23]	Switzerland, ED	Prospective interventional feasibility study	Adult ED patients with traumatic or non-traumatic pain; n = 52	Virtual reality simulation	Pre-post within-subject comparison	NRS pain, NRS anxiety, user experience	Pain and anxiety decreased after VR; satisfaction and tolerability were high	ROBINS-I
Bosso et al., 2023 [24]	Switzerland, ED	Randomized controlled study	Adults undergoing minor ED procedures; n = 117	3D virtual reality	2D virtual reality	Pain and anxiety during minor procedures	3D VR did not significantly reduce average procedural pain or anxiety compared with 2D VR, although the intervention was feasible and telepresence was higher	RoB 2
Ko et al., 2024 [25]	Hong Kong, ED	Pilot randomized controlled trial	Adults undergoing wound closure; n = 80	Virtual reality plus standard care	Standard care	Anxiety, pain, vital signs, satisfaction, additional local anesthesia	VR reduced anxiety, pain, physiological stress markers, and additional local anesthesia requests	RoB 2
Goktas and Avci, 2023 [26]	Turkey, pediatric ED	Single-blinded randomized controlled trial	Children aged 7 to 12 undergoing invasive procedures; n = 144	Kaleidoscope, music, or VR distraction	Standard invasive procedure	Wong-Baker pain scale, Children’s Anxiety Meter-State, Child Medical Fear Scale	All distraction methods reduced pain, anxiety, and medical fear; VR showed the strongest anxiety effect	RoB 2
Dusek et al., 2024 [27]	USA, three EDs	Multicenter feasibility randomized controlled trial	Adults with acute non-emergent pain ≥4/10; n = 165	Acupuncture	Usual care	Pain, anxiety, recruitment, retention, acceptability	Acupuncture was feasible and acceptable, with greater pre-post reductions in pain and anxiety than usual care	RoB 2
Burns et al., 2019 [28]	USA, urban ED	Retrospective quality improvement study	Adults offered acupuncture; n = 379	Acupuncture	Pre-post comparison	Pain, stress, anxiety, nausea	Acupuncture was associated with reductions in pain, stress, anxiety, and nausea	ROBINS-I
Tupetz et al., 2025 [29]	USA, ED	Qualitative evaluation embedded in pragmatic trial	ED patients receiving acupuncture for acute musculoskeletal pain; n = 28 interviews	Acupuncture experience	Not applicable	Patient experience, acceptability, perceived pain/stress improvement	Participants described positive experiences, acceptability, and perceived improvements in pain, stress, anxiety, and sleep	CASP qualitative
Belland et al., 2017 [30]	USA, geriatric ED	Randomized pilot study	Older ED adults >65 years; n = 35	Music listening	Routine care	STAI anxiety	Music listening reduced anxiety compared with control	RoB 2
Weiland et al., 2011 [31]	Australia, ED	Randomized controlled trial	Adult ED patients; n = 170 completed baseline anxiety assessment	Purpose-designed music/sound compositions	Ambient noise or headphones only	STAI anxiety	Selected sound compositions reduced state anxiety by approximately 10% to 15%	RoB 2
Ferraz-Torres et al., 2023 [32]	Spain, pediatric emergency care	Quasi-experimental study	Children aged 2 to 15 undergoing venipuncture; n = 458	Virtual reality distraction	Standard care	FLACC, NRS pain, Childhood Anxiety Scale, parental behavioral response	VR reduced pain and anxiety and improved parental or companion behavioral response	ROBINS-I
O’Sullivan et al., 2023 [33]	Pediatric ED	Prospective interventional cohort pilot study	Children aged 8 to 15 undergoing needle-related procedures; n = 22	VR-delivered hypnotherapy	Pre-post comparison	Visual Analog Anxiety Scale, Color Analog Pain Scale, satisfaction	VR hypnotherapy reduced procedural anxiety for many participants; pain findings were less consistent	ROBINS-I
Chan et al., 2019 [34]	Australia, ED and outpatient pathology	Two randomized clinical trials	Children aged 4 to 11 undergoing venous needle procedures; ED arm n = 123, with 64 assigned to VR and 59 to standard care	Virtual reality distraction	Standard care	Faces Pain Scale-Revised, adverse events	In the ED arm, VR reduced pain compared with standard care and was safe	RoB 2
Dumoulin et al., 2019 [35]	Canada, pediatric ED	Randomized controlled trial	Children/adolescents aged 8 to 17 undergoing needle procedures; n = 59	VR distraction	Television or child life distraction	VAS pain intensity, fear of pain, satisfaction	VR produced greater reduction in fear of pain and high satisfaction; pain intensity reduction was not superior	RoB 2
Sikka et al., 2019 [36]	USA, urban ED	Prospective cohort study	ED patients with pain score ≥3; n = 100 enrolled, 67 completed VR	Immersive VR applications	Pre-post comparison	Pain, anger, anxiety scores	VR was feasible and associated with reductions in pain, anger, and anxiety among completers	ROBINS-I
Osmanlliu et al., 2021 [37]	Canada, pediatric ED	Pilot pragmatic randomized controlled trial	Children undergoing IV procedures; n = 63	VR distraction plus standard care	Standard care	Pain, distress, memory of pain, satisfaction, adverse effects	VR was feasible and acceptable; pain scores were similar, but distress and memory-of-pain outcomes favored VR descriptively	RoB 2
Canares et al., 2021 [38]	USA, pediatric ED	Pilot randomized controlled trial	Children/young adults aged 7 to 22 undergoing venipuncture; n = 55 analyzed	Child life-supported VR	Child life support without VR; external reference group	CAMPIS-SF coping/distress, pain, anxiety, cybersickness	VR was feasible and improved observed coping; pain and distress differences were not consistently significant	RoB 2
Schlechter et al., 2021 [39]	USA, pediatric ED	Prospective randomized comparison study	Children aged 4 to 17 requiring IV placement; n = 116 enrolled, 115 analyzed	VR distraction	Standard distraction/non-VR care	IV success, pain, anxiety, tolerance	VR was well tolerated and showed similar efficacy to standard distraction techniques during IV placement	RoB 2
Butt et al., 2022 [40]	USA, pediatric ED	Randomized controlled trial	Adolescent pediatric ED patients; n = 110	Mindfulness-based VR program, Take-Pause	Passive distraction/control care	Anxiety, stress, procedural experience	Mindfulness-based VR reduced anxiety more than passive distraction, while pain-related secondary outcomes were not clearly different between groups	RoB 2

Synthesis of Key Findings

Across the 25 included studies, the overall pattern of evidence suggested that non-pharmacological interventions were more consistently associated with short-term improvements in emotional and experiential outcomes than with uniform reductions in pain intensity [16-40]. Improvements were most consistently reported for anxiety, fear, distress, coping, comfort, satisfaction, procedural tolerance, and patient or caregiver experience. Pain intensity outcomes were more variable. This variability appeared related to differences in intervention type, comparator, patient population, procedural versus non-procedural pain context, timing of assessment, outcome measurement tools, and study design. Because of these clinical and methodological differences, a pooled quantitative synthesis was not performed, and findings were summarized narratively by intervention category and outcome domain.

Music and sound-based interventions were evaluated in nine studies [16-22,30,31]. These interventions were not uniform and included therapist-delivered music therapy, self-selected music, smartphone-based music applications, live environmental music therapy, prerecorded music, and purpose-designed sound compositions. This variation in delivery method may influence clinical effects, acceptability, and feasibility. Overall, music and sound-based interventions showed the most consistent favorable pattern for anxiety, stress, comfort, and patient experience, while pain outcomes were mixed but often favorable. Randomized or controlled studies in emergency department patients with acute pain complaints or musculoskeletal back pain reported reductions in both pain and anxiety [16,17]. A smartphone-based music app produced modest reductions in pain and anxiety among emergency department observation-unit patients [18]. In wound-suturing studies, music appeared more consistently associated with anxiety reduction than with statistically robust pain reduction [19,20]. Studies focused on satisfaction and care experience suggested that music therapy may be acceptable and may improve perceived emergency department service quality, although formal satisfaction outcomes were not uniformly significant [21,22]. Among older adults and general emergency department patients, music or sound interventions reduced anxiety even when pain was not the primary target [30,31].

Virtual reality and distraction-based interventions formed the largest intervention group and were evaluated in 13 studies [23-26,32-40]. The strongest pattern of evidence was seen in pediatric and procedural contexts, particularly venipuncture, intravenous cannulation, wound closure, and other needle-related procedures. In adult emergency department populations, virtual reality was generally feasible and acceptable, with several studies reporting reductions in pain or anxiety after exposure [23,25,36]. Adult procedural studies suggested potential benefits for anxiety, pain, physiological stress markers, satisfaction, and additional local anesthetic use, although interpretation was limited in some studies by pilot designs or active comparators [24,25]. Pediatric studies generally supported the feasibility and acceptability of virtual reality or distraction approaches and showed more consistent benefits for anxiety, fear, coping, and distress than for pain intensity [26,32-40]. Pain findings were mixed, with some trials reporting lower child-reported pain and others finding similar pain scores compared with standard distraction or child life support.

Outcome measurement varied substantially across the included studies, which affected comparability and certainty of interpretation. Pain was assessed using tools such as numerical rating scales, visual analog scales, Faces Pain Scale-Revised, Wong-Baker pain scale, FLACC, and CHEOPS. Anxiety, fear, distress, and coping were assessed using tools such as State-Trait Anxiety Inventory (STAI), STAI-6, Children’s Anxiety Meter-State, Child Medical Fear Scale, CAMPIS-SF, visual analog anxiety scales, behavioral observation tools, and study-specific Likert or satisfaction scales [16-40]. Validated outcome measures provide stronger interpretive confidence than non-validated or study-specific scales, and this variation in measurement tools contributed to heterogeneity across studies.

Acupuncture-based interventions were evaluated in three studies [27-29]. The multicenter ACUITY feasibility randomized trial reported that emergency department acupuncture was feasible and acceptable under study conditions and was associated with greater pre-post reductions in pain and anxiety than usual care [27]. A retrospective quality improvement study reported reductions in pain, stress, anxiety, and nausea after emergency department acupuncture [28]. The qualitative evaluation added patient-experience evidence, showing that patients with acute musculoskeletal pain generally perceived emergency department acupuncture as acceptable and convenient, with perceived improvements in pain, stress, anxiety, and sleep [29]. These findings should be interpreted cautiously because feasibility in routine emergency department practice may depend on trained personnel, patient selection, available space, timing, crowding, and integration with standard emergency department workflow.

Overall, the direction of evidence was more consistently favorable for emotional and experiential outcomes, especially anxiety, fear, distress, coping, comfort, satisfaction, and patient experience, than for pain intensity. Music and sound-based interventions showed a more consistent favorable pattern for anxiety than for pain intensity, but conclusions about broad feasibility should remain cautious because some evidence came from pilot, service evaluation, or context-specific designs. Virtual reality and distraction interventions showed the most consistent favorable findings in pediatric procedural anxiety, fear, coping, and distress, although analgesic effects were heterogeneous. Acupuncture showed preliminary favorable findings for adult acute pain and associated distress, but the evidence was limited by feasibility, quality improvement, and qualitative designs. Therefore, current evidence suggests possible short-term benefit for pain-related distress and patient experience in selected emergency department contexts, while definitive conclusions about analgesic effectiveness remain limited.

Risk of Bias Assessment

Risk of bias was assessed according to study design using the tools described in the Methods. Randomized and pilot randomized trials were assessed using RoB 2, non-randomized prospective, quasi-experimental, before-after, feasibility, service evaluation, and quality improvement studies were assessed using ROBINS-I, and the qualitative study was assessed using the CASP qualitative checklist. Because the included studies differed substantially in design and methodological purpose, risk-of-bias judgments were interpreted according to the appropriate tool rather than as a single pooled quality rating.

The main risk-of-bias judgments and key concerns for all included studies are summarized in Table 4.

**Table 4:** Risk-of-bias assessment by study design and tool CASP, Critical Appraisal Skills Programme; ROBINS-I, Risk Of Bias In Non-randomized Studies of Interventions; RoB 2, Cochrane Risk of Bias 2 tool.

Study	Tool	Overall judgment	Main concerns
Angkoontassaneeyarat et al., 2025 [16]	RoB 2	Some concerns	Randomization by selected study days, lack of participant blinding, self-reported outcomes
Goldfine et al., 2025 [17]	RoB 2	Some concerns	Pilot sample, baseline imbalance in pain catastrophizing, subjective outcomes
Chai et al., 2020 [18]	ROBINS-I	Moderate	Non-randomized/prospective design, possible selection bias, no usual-care control arm
Hedayati et al., 2023 [19]	RoB 2	Some concerns	Limited blinding, small sample, subjective pain/anxiety measurement
Bakhshandeh et al., 2024 [20]	RoB 2	Some concerns	Blinding unclear, pediatric behavioral pain measurement, limited methodological detail
Mandel et al., 2019 [21]	ROBINS-I	Serious	Service evaluation design, selection bias, confounding, satisfaction subgroup analysis
Ettenberger et al., 2025 [22]	RoB 2	Some concerns	Pragmatic design, blinding unlikely, subjective patient/caregiver outcomes
Birrenbach et al., 2022 [23]	ROBINS-I	Serious	Single-arm pre-post feasibility design, no parallel control, regression-to-the-mean risk
Bosso et al., 2023 [24]	RoB 2	Some concerns	Active comparator, blinding challenges, subjective pain/anxiety outcomes
Ko et al., 2024 [25]	RoB 2	Some concerns	Pilot design, no participant blinding, subjective outcomes despite objective physiological measures
Goktas and Avci, 2023 [26]	RoB 2	Some concerns	Single-blind design, subjective child-reported outcomes, multiple intervention arms
Dusek et al., 2024 [27]	RoB 2	Some concerns	Feasibility trial, not powered for definitive efficacy, blinding unlikely
Burns et al., 2019 [28]	ROBINS-I	Serious	Retrospective quality improvement data, no control group, confounding by co-interventions
Tupetz et al., 2025 [29]	CASP qualitative	Methodological limitations identified	Interview-based data, possible selection bias toward acupuncture recipients with favorable experiences
Belland et al., 2017 [30]	RoB 2	Some concerns	Small pilot sample, blinding not feasible, anxiety-only primary outcome
Weiland et al., 2011 [31]	RoB 2	Some concerns	Randomized design with partial blinding, self-reported outcome, short-term follow-up
Ferraz-Torres et al., 2023 [32]	ROBINS-I	Moderate	Quasi-experimental design, allocation not fully randomized, subjective and behavioral outcomes
O’Sullivan et al., 2023 [33]	ROBINS-I	Serious	Small uncontrolled pilot cohort, no comparator group, feasibility/implementation focus
Chan et al., 2019 [34]	RoB 2	Some concerns	Two settings, ED subgroup relevance, blinding not feasible
Dumoulin et al., 2019 [35]	RoB 2	Some concerns	Small sample, active comparators, subjective pain/fear ratings
Sikka et al., 2019 [36]	ROBINS-I	Serious	Prospective cohort without control group, incomplete intervention completion, selection/tolerance bias
Osmanlliu et al., 2021 [37]	RoB 2	Some concerns	Pilot pragmatic trial, feasibility primary focus, subjective outcomes, small sample
Canares et al., 2021 [38]	RoB 2	Some concerns	Pilot design, small randomized groups, external reference group, feasibility outcomes
Schlechter et al., 2021 [39]	RoB 2	Some concerns	Procedural feasibility and IV success primary focus, subjective secondary pain/anxiety outcomes
Butt et al., 2022 [40]	RoB 2	Some concerns	Pediatric ED anxiety/stress focus, blinding not feasible, subjective outcomes

The domain-level RoB 2 assessment for randomized and pilot randomized studies is presented in Table 5.

**Table 5:** RoB 2 domain-level assessment for randomized and pilot randomized studies RoB 2 judgments were categorized as low risk, some concerns, or high risk. The most frequent concerns involved lack of participant blinding, subjective self-reported outcomes, pilot sample size, and short follow-up.

Study	Randomization process	Deviations from intended interventions	Missing outcome data	Measurement of outcome	Selection of reported result	Overall judgment
Angkoontassaneeyarat et al., 2025 [16]	Some concerns	Some concerns	Low risk	Some concerns	Low risk	Some concerns
Goldfine et al., 2025 [17]	Some concerns	Some concerns	Low risk	Some concerns	Low risk	Some concerns
Hedayati et al., 2023 [19]	Some concerns	Some concerns	Low risk	Some concerns	Low risk	Some concerns
Bakhshandeh et al., 2024 [20]	Some concerns	Some concerns	Some concerns	Some concerns	Some concerns	Some concerns
Ettenberger et al., 2025 [22]	Low risk	Some concerns	Low risk	Some concerns	Low risk	Some concerns
Bosso et al., 2023 [24]	Low risk	Some concerns	Low risk	Some concerns	Low risk	Some concerns
Ko et al., 2024 [25]	Some concerns	Some concerns	Low risk	Some concerns	Low risk	Some concerns
Goktas and Avci, 2023 [26]	Low risk	Some concerns	Low risk	Some concerns	Low risk	Some concerns
Dusek et al., 2024 [27]	Some concerns	Some concerns	Low risk	Some concerns	Low risk	Some concerns
Belland et al., 2017 [30]	Some concerns	Some concerns	Some concerns	Some concerns	Low risk	Some concerns
Weiland et al., 2011 [31]	Low risk	Some concerns	Low risk	Some concerns	Low risk	Some concerns
Chan et al., 2019 [34]	Low risk	Some concerns	Low risk	Some concerns	Some concerns	Some concerns
Dumoulin et al., 2019 [35]	Some concerns	Some concerns	Low risk	Some concerns	Low risk	Some concerns
Osmanlliu et al., 2021 [37]	Some concerns	Some concerns	Some concerns	Some concerns	Low risk	Some concerns
Canares et al., 2021 [38]	Some concerns	Some concerns	Some concerns	Some concerns	Some concerns	Some concerns
Schlechter et al., 2021 [39]	Some concerns	Some concerns	Low risk	Some concerns	Low risk	Some concerns
Butt et al., 2022 [40]	Some concerns	Some concerns	Some concerns	Some concerns	Low risk	Some concerns

The domain-level ROBINS-I assessment for non-randomized, quasi-experimental, feasibility, service evaluation, and quality improvement studies is presented in Table 6.

**Table 6:** ROBINS-I domain-level assessment for non-randomized, feasibility, service evaluation, and quality improvement studies ROBINS-I judgments were categorized as low, moderate, serious, critical, or no information. Main concerns included absence of parallel control groups, possible selection bias, confounding by usual emergency department analgesia or procedural care, and pre-post designs. ROBINS-I, Risk Of Bias In Non-randomized Studies of Interventions

Study	Confounding	Selection of participants	Classification of interventions	Deviations from intended interventions	Missing data	Measurement of outcomes	Selection of reported result	Overall judgment
Chai et al., 2020 [18]	Moderate	Moderate	Low	Moderate	Moderate	Moderate	Moderate	Moderate
Mandel et al., 2019 [21]	Serious	Serious	Moderate	Serious	Moderate	Moderate	Moderate	Serious
Birrenbach et al., 2022 [23]	Serious	Serious	Low	Moderate	Moderate	Moderate	Moderate	Serious
Burns et al., 2019 [28]	Serious	Serious	Low	Serious	Moderate	Moderate	Moderate	Serious
Ferraz-Torres et al., 2023 [32]	Moderate	Moderate	Low	Moderate	Moderate	Moderate	Moderate	Moderate
O’Sullivan et al., 2023 [33]	Serious	Serious	Low	Moderate	Moderate	Moderate	Moderate	Serious
Sikka et al., 2019 [36]	Serious	Serious	Low	Moderate	Serious	Moderate	Moderate	Serious

 The CASP qualitative appraisal for the included qualitative study is presented in Table 7.

**Table 7:** CASP qualitative appraisal CASP qualitative appraisal findings were summarized narratively because CASP does not assign a formal overall risk-of-bias category. Judgments were recorded as yes, no, or cannot tell when applicable. CASP, Critical Appraisal Skills Programme

Study	Clear aims	Appropriate methodology	Appropriate design	Recruitment strategy	Data collection	Researcher reflexivity	Ethical issues	Data analysis	Findings clear	Overall appraisal
Tupetz et al., 2025 [29]	Yes	Yes	Yes	Cannot tell	Yes	Cannot tell	Cannot tell	Yes	Yes	Methodological limitations identified

**Figure 2:**
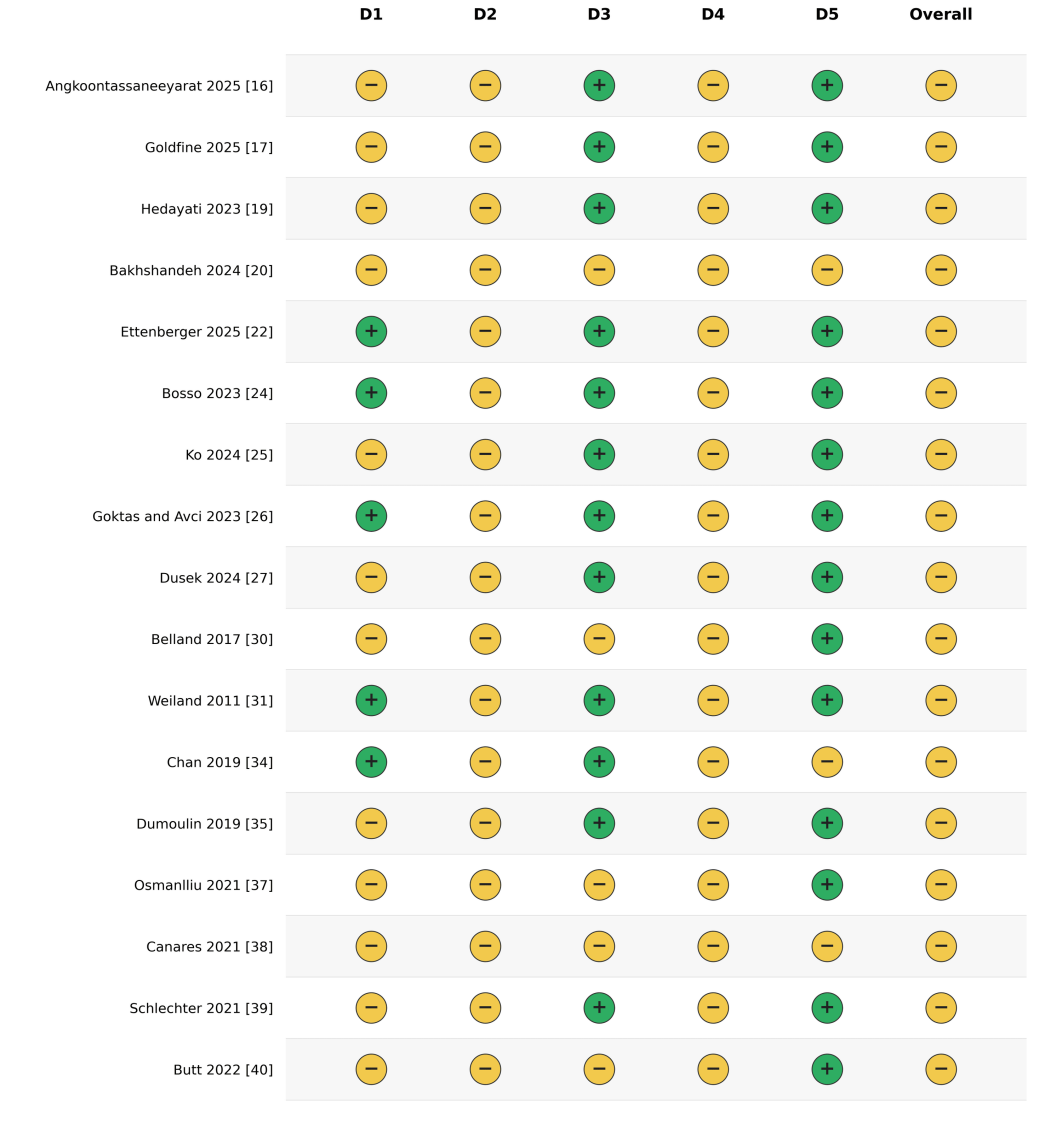
RoB 2 study-level traffic-light plot Traffic-light plot showing domain-level RoB 2 judgments for randomized and pilot randomized studies. Green circles with a plus sign indicate low risk of bias, yellow circles with a minus sign indicate some concerns, and red circles with a cross indicate high risk of bias. No included randomized or pilot randomized study was judged as high risk overall. RoB 2, Cochrane Risk of Bias 2 tool; D1, bias arising from the randomization process; D2, bias due to deviations from intended interventions; D3, bias due to missing outcome data; D4, bias in measurement of the outcome; D5, bias in selection of the reported result.

 Study-level RoB 2 judgments for randomized and pilot randomized studies are shown in Figure 2.

**Figure 3:**
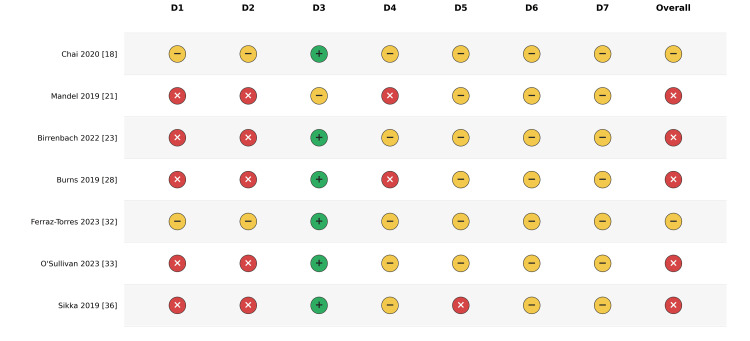
ROBINS-I study-level traffic-light plot Traffic-light plot showing domain-level ROBINS-I judgments for non-randomized, quasi-experimental, feasibility, service evaluation, and quality improvement studies. Green circles with a plus sign indicate low risk of bias, yellow circles with a minus sign indicate moderate risk of bias, and red circles with a cross indicate serious risk of bias. No study was judged as critical risk of bias or as having no information. ROBINS-I, Risk Of Bias In Non-randomized Studies of Interventions; D1, bias due to confounding; D2, bias in selection of participants; D3, bias in classification of interventions; D4, bias due to deviations from intended interventions; D5, bias due to missing data; D6, bias in measurement of outcomes; D7, bias in selection of the reported result.

Study-level ROBINS-I judgments for non-randomized, quasi-experimental, feasibility, service evaluation, and quality improvement studies are shown in Figure 3.

**Figure 4:**
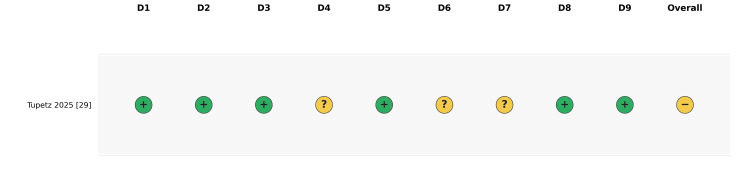
CASP qualitative appraisal traffic-light plot Traffic-light plot showing CASP qualitative appraisal judgments for the included qualitative study. For individual CASP domains, green circles with a plus sign indicate yes, yellow circles with a minus sign indicate cannot tell, and red circles with a cross indicate no. For the overall appraisal column, the yellow circle indicates methodological limitations identified rather than cannot tell. CASP, Critical Appraisal Skills Programme; D1, clear aims; D2, appropriate qualitative methodology; D3, appropriate research design; D4, recruitment strategy; D5, data collection; D6, researcher reflexivity; D7, ethical issues; D8, data analysis; D9, clear statement of findings.

The CASP qualitative appraisal for the included qualitative study is shown in Figure 4.

The risk-of-bias tables and traffic-light plots show that methodological concerns were common across the included evidence. Among randomized and pilot randomized studies assessed with RoB 2, all studies were judged as having some concerns overall rather than low risk of bias. The most frequent concerns were related to deviations from intended interventions and measurement of outcomes. These concerns were expected in part because participant blinding is difficult or not feasible for behavioral and sensory interventions such as music, virtual reality, distraction, and acupuncture. Several studies also relied on subjective outcomes, including pain, anxiety, fear, satisfaction, and procedural experience, which increased concerns about outcome measurement.

For randomized and pilot randomized studies, missing outcome data and selection of the reported result were generally less problematic than blinding and outcome measurement. However, some studies had additional concerns related to small pilot samples, short follow-up windows, baseline imbalance, unclear randomization procedures, or feasibility-focused designs that were not powered for definitive efficacy. Therefore, the randomized evidence was useful for identifying possible short-term effects but remained insufficient for strong conclusions about definitive analgesic effectiveness or sustained benefit.

Among non-randomized, quasi-experimental, feasibility, service evaluation, and quality improvement studies assessed with ROBINS-I, overall judgments were moderate or serious. The most frequent concerns involved confounding, selection of participants, absence of parallel control groups, pre-post designs, and possible influence of usual emergency department analgesia or procedural care. These limitations are important because observed pre-post improvements may reflect regression to the mean, concurrent analgesic treatment, patient selection, procedural factors, staff interaction, or other aspects of emergency department care rather than the non-pharmacological intervention alone.

The qualitative acupuncture study provided useful information about patient experience, acceptability, and perceived benefit, but it did not directly estimate intervention effectiveness. CASP appraisal identified methodological limitations, particularly because some information about recruitment strategy, researcher reflexivity, and ethical considerations was unclear. Therefore, the qualitative evidence was interpreted as supportive contextual evidence rather than causal evidence.

Overall, the risk-of-bias assessment supports cautious interpretation of the review findings. The included studies are more informative for feasibility, acceptability, anxiety or distress outcomes, coping, comfort, satisfaction, and patient experience than for definitive analgesic effectiveness, sustained benefit, or analgesic-sparing effects. These risk-of-bias concerns were considered when interpreting the synthesis and when wording the Discussion and Conclusion.

Discussion

Summary of Main Findings

This systematic review synthesized evidence on non-pharmacological interventions for pain-related anxiety, fear, distress, coping, comfort, satisfaction, patient experience, and pain intensity in emergency department settings. Across the included studies, findings were more consistently favorable for emotional and experiential outcomes than for pain intensity [16-40]. Reported improvements were most frequent for anxiety, fear, distress, coping, comfort, satisfaction, procedural tolerance, and patient or caregiver experience, whereas pain intensity findings were more variable.

The main intervention categories identified were music and sound-based interventions, virtual reality and distraction-based interventions, and acupuncture-based interventions. These approaches should not be interpreted as a single uniform intervention group because they differed substantially in delivery method, intensity, comparator, patient population, clinical context, and outcome measurement. The overall certainty of evidence remains limited because many studies were small, pilot, feasibility-based, non-randomized, short-term, or at risk of bias. Therefore, the findings suggest possible short-term benefit in selected emergency department contexts, but they do not establish definitive analgesic effectiveness.

Interpretation by Intervention Type

Music and sound-based interventions were evaluated across several emergency department populations and settings, including adult, pediatric, older adult, and waiting-room contexts [16-22,30,31]. These interventions included therapist-delivered music therapy, self-selected music, smartphone-based music applications, live or prerecorded music, and purpose-designed sound compositions. Because these delivery methods differ in patient engagement, staffing needs, intervention intensity, and feasibility, their effects should not be assumed to be equivalent.

Overall, music and sound-based interventions showed a more consistent favorable pattern for anxiety, stress, comfort, and patient experience than for pain intensity. Some randomized or controlled studies reported reductions in both pain and anxiety, particularly when music was used alongside standard care or when patients selected or engaged with the intervention [16-18]. In procedural wound care, music appeared more consistently associated with anxiety reduction than with statistically robust pain reduction, although pediatric suturing data suggested possible improvement in both child pain and parental anxiety [19,20]. Music interventions were also reported as acceptable in several studies, but conclusions about broad feasibility or scalability should remain cautious because some evidence came from pilot, service evaluation, or context-specific designs [21,22,30,31].

Virtual reality and distraction-based interventions represented the largest and most heterogeneous intervention group. The most consistent favorable findings were observed in pediatric procedural contexts, particularly venipuncture, intravenous cannulation, wound closure, and other needle-related procedures [23-26,32-40]. In adult emergency department settings, virtual reality appeared feasible and acceptable in selected studies, with some reporting short-term reductions in pain or anxiety after exposure [23,25,36]. However, interpretation was limited by pilot designs, active comparators, small samples, and variation in outcome measurement [24,25].

In pediatric studies, virtual reality and distraction approaches more consistently favored anxiety, fear, coping, and distress outcomes than pain intensity outcomes [26,32-40]. Some trials reported lower child-reported pain, while others found pain scores similar to standard distraction or child-life support. These findings suggest that virtual reality and distraction may be most relevant for emotional regulation, coping, and procedural tolerance, but they do not demonstrate a consistent analgesic effect across settings.

Acupuncture formed a smaller evidence group. The available studies suggested that emergency department acupuncture was feasible and acceptable under selected study conditions and was associated with short-term reductions in pain, anxiety, stress, or nausea in some adult acute pain contexts [27-29]. However, this evidence remains preliminary because it included feasibility, quality improvement, and qualitative designs. Acupuncture also requires trained practitioners, appropriate patient selection, suitable space, and workflow integration. Therefore, its feasibility and effectiveness in routine emergency department practice remain uncertain and may vary substantially by local resources and clinical context.

Comparison With Previous Literature

The findings of this review are broadly consistent with previous reviews suggesting that non-pharmacological interventions may reduce pain or anxiety in selected emergency or procedural-care contexts, while also emphasizing substantial heterogeneity and limited certainty [6-10]. Previous reviews of emergency department non-pharmacological pain interventions and pediatric procedural pain have reported variability in populations, procedures, intervention delivery, comparators, and outcome tools [6,7]. Reviews of music-based interventions have suggested possible reductions in anxiety and pain, but have also highlighted differences in intervention format, setting, and outcome assessment [8]. Similarly, reviews of virtual reality have reported potential benefits for procedural pain and anxiety, particularly in pediatric or procedural settings, while noting small trials, heterogeneous comparators, and inconsistent pain effects [9,10].

This review adds to prior work by synthesizing pain intensity together with pain-related emotional distress, coping, comfort, satisfaction, patient experience, feasibility, and analgesic-related outcomes across several emergency department intervention categories. The findings support a cautious interpretation: emotional and experiential outcomes were more consistently favorable than pain intensity outcomes, while evidence for clinically meaningful analgesic reduction, sustained benefit, or opioid-sparing effect remains insufficient.

Mechanisms and Clinical Relevance

Non-pharmacological interventions may plausibly influence pain-related experience through emotional, cognitive, and behavioral pathways. Acute pain in the emergency department may be intensified by anxiety, fear, uncertainty, attentional focus, and perceived loss of control. Interventions such as music, virtual reality, distraction, and acupuncture may help by redirecting attention, reducing anxiety, supporting coping, increasing perceived control, or making procedures more tolerable. These mechanisms should be interpreted as plausible explanations rather than proven causal pathways because most included studies were not designed to test mechanisms directly [16-40].

Several study-level findings are consistent with this interpretation. Goldfine et al. reported anxiety reduction after self-selected music, suggesting that emotional improvement may accompany pain improvement in some patients [17]. Chai et al. indicated that patients with higher baseline pain, anxiety, or catastrophizing may derive greater benefit from a music-based intervention [18]. Canares et al. showed that virtual reality supported observed coping during venipuncture, even when pain and distress differences were not consistently significant [38]. These findings suggest that pain-score reduction should not be the only outcome considered. In emergency care, improvements in coping, fear, cooperation, tolerance of procedures, or patient experience may still be meaningful, particularly in pediatric and procedural contexts [26,35,37,38].

Patient Experience and Acceptability

Patient experience is an important outcome in emergency pain management. Several studies reported that patients, caregivers, or staff valued non-pharmacological interventions even when pain outcomes were modest or mixed. Music interventions were described as acceptable and easy to use in several studies, with possible benefits for perceived emergency department service quality and staff or patient experience [16,18,21]. Virtual reality studies also commonly reported feasibility, acceptability, or satisfaction among patients, parents, or healthcare providers [23,25,37,38]. These findings suggest that non-pharmacological interventions may improve aspects of the subjective care experience through reassurance, distraction, comfort, perceived support, and a greater sense of control, especially in crowded or stressful clinical environments.

Analgesic and Opioid-Sparing Outcomes

The evidence for analgesic reduction or opioid-sparing effects remains insufficient. Although several studies were motivated by the need for safer or supportive approaches to emergency pain care, few were designed to evaluate analgesic use, rescue medication, or opioid exposure as primary endpoints. Chai et al. included patients with opioid orders but did not establish opioid reduction as a primary outcome [18]. Ko et al. reported fewer requests for additional local anesthesia during wound closure, but this does not directly establish systemic analgesic or opioid-sparing benefit [25]. Burns et al. reported improvements in pain and distress after acupuncture, but the quality improvement design could not isolate analgesic-sparing effects from other aspects of care [28]. Therefore, analgesic-sparing and opioid-sparing effects should be treated as future research questions rather than established findings of this review.

Measurement Issues and Evidence Certainty

Outcome heterogeneity limited direct comparison across studies. Pain was measured using numerical rating scales, visual analog scales, Faces Pain Scale-Revised, Wong-Baker Faces Pain Rating Scale, CHEOPS, FLACC, and behavioral observation measures. Anxiety, fear, distress, and coping were measured using STAI, STAI-6, visual analog anxiety scales, Children’s Anxiety Meter-State, Child Medical Fear Scale, CAMPIS-SF, and other procedure-specific or study-specific tools [16-20,22,26,32,34,35,38]. These instruments capture related but non-identical constructs, which reduces comparability and limits the appropriateness of pooled quantitative analysis. Panic-like distress was not assessed using validated panic-specific tools, despite being part of the broader review scope.

Risk of bias further limits certainty. Many studies were small, pilot, pragmatic, feasibility-based, single-arm, or non-randomized. Blinding was usually not feasible, and outcomes were often self-reported and measured shortly after the intervention. Non-randomized studies were vulnerable to selection bias, confounding by co-interventions, regression to the mean, and lack of parallel controls [18,21,23,28,29,32,33,36]. Therefore, certainty is greater for feasibility, acceptability, short-term anxiety or distress outcomes, coping, and patient experience than for definitive analgesic effectiveness, opioid-sparing effects, or sustained post-discharge benefit.

Implementation and Future Research

Implementation should be considered in relation to the intervention and clinical context. Music-based approaches may be relatively low-burden in some settings, especially when delivered through prerecorded, self-selected, or app-based formats, although live music therapy requires trained personnel and dedicated service models [18,21,22]. Virtual reality may be suitable for selected adult procedures and pediatric procedural distress, but it requires equipment, cleaning protocols, staff support, patient screening, and monitoring for intolerance or cybersickness [37,38]. Acupuncture may be considered only in settings where trained practitioners, appropriate patient selection, and workflow capacity are available [27,29]. Future studies should evaluate clinical effects together with staffing burden, infection control, cost, cultural acceptability, equity of access, and integration into routine emergency department workflows.

Future research should move beyond small pilot and feasibility studies toward adequately powered, multicenter, pragmatic trials. These trials should use standardized core outcomes for pain, anxiety, fear, distress, coping, satisfaction, analgesic use, and patient experience. They should also evaluate clinically meaningful pain relief, rescue medication, morphine milligram equivalents, additional local anesthetic use, adverse effects, post-discharge outcomes, and implementation feasibility. Direct comparisons between active interventions, such as music versus virtual reality, virtual reality versus child-life support, or acupuncture versus usual care with standardized analgesic protocols, would help clarify which interventions may be most appropriate for specific patient groups and emergency department contexts.

Limitations

This review has several limitations. The protocol was not registered, and a formal meta-analysis was not performed because of substantial heterogeneity in populations, interventions, comparators, outcome instruments, measurement timing, and study designs. Incomplete and inconsistent reporting of quantitative outcome data also limited the feasibility of standardized effect-size calculation or subgroup meta-analysis. A formal GRADE assessment was not performed, so certainty of evidence was interpreted narratively.

Although the search used bibliographic databases, semantic search platforms, publisher webpages, DOI checking, and citation tracking, unpublished studies, local service evaluations, and non-indexed interventions may have been missed. Because the synthesis relied on reports with sufficient extractable information in English, relevant data available only in other languages or insufficiently indexed sources may have been underrepresented. Publication bias and selective outcome reporting could not be formally assessed. Selective reporting is particularly relevant because studies with favorable findings for pain, anxiety, satisfaction, or feasibility may be more likely to be published or reported in detail.

The underlying evidence base also had limitations. Many studies were small, short-term, pilot, feasibility, quasi-experimental, single-arm, or quality improvement studies. Several outcomes were subjective and measured immediately after the intervention. Older adults, high-risk emergency populations, and patients with cognitive or communication barriers were underrepresented. Panic-like distress was not measured using validated panic-specific instruments, and analgesic or opioid-sparing outcomes were inconsistently reported. These limitations mean that the findings should be interpreted as suggestive rather than definitive evidence for non-pharmacological interventions in emergency pain care.

## Conclusions

Current evidence suggests possible short-term benefits from non-pharmacological interventions for pain-related anxiety, fear, distress, coping, comfort, satisfaction, and patient experience in selected emergency department contexts, but certainty remains limited. Effects on pain intensity were more variable, and the available evidence is insufficient to determine definitive analgesic effectiveness, sustained benefit, or analgesic-sparing effects. Music and sound-based interventions, virtual reality and distraction-based interventions, and acupuncture suggested possible benefit in selected settings, but findings were limited by heterogeneity, small samples, short follow-up, subjective outcomes, and risk-of-bias concerns.

Future adequately powered trials should use standardized outcome measures, assess clinically meaningful pain and anxiety outcomes, evaluate rescue medication and opioid-sparing outcomes, and test implementation feasibility across diverse emergency department settings.

## Appendices

The completed PRISMA 2020 checklist is presented in Table 8.

**Table 8:** PRISMA 2020 checklist PRISMA, Preferred Reporting Items for Systematic Reviews and Meta-Analyses.

Item	Topic	PRISMA 2020 item	Location in revised manuscript	Status or note
1	Title	Identify the report as a systematic review.	Title	Addressed
2	Abstract	Provide a structured summary of background, objectives, eligibility criteria, information sources, synthesis methods, results, limitations, and implications.	Abstract	Addressed as a single continuous Cureus-style abstract
3	Rationale	Describe the rationale for the review in the context of existing knowledge.	Introduction and Background	Addressed
4	Objectives	Provide an explicit statement of the objective or question.	End of Introduction and Research Question section	Addressed
5	Eligibility criteria	Specify inclusion and exclusion criteria and how studies were grouped.	Methods, Eligibility Criteria and Table 1	Addressed
6	Information sources	Specify all databases, registers, websites, organizations, reference lists, and other sources searched, with the date when each was last searched.	Methods, Information Sources and Search Strategy	Addressed
7	Search strategy	Present the full search strategies for all databases, registers, and websites, including filters and limits.	Methods, Table 2 and supplementary search details	Addressed
8	Selection process	Specify methods used to decide whether a study met inclusion criteria, including reviewers and automation tools.	Methods, Study Selection	Addressed
9	Data collection process	Specify methods used to collect data, including reviewers, piloting, and how missing data were handled.	Methods, Data Extraction	Addressed
10a	Data items, outcomes	List and define all outcomes for which data were sought.	Methods, Table 1 and Data Extraction	Addressed
10b	Data items, other variables	List and define all other variables for which data were sought.	Methods, Data Extraction	Addressed
11	Study risk of bias assessment	Specify methods and tools used to assess risk of bias.	Methods, Risk-of-Bias Assessment and Supplementary File 2	Addressed
12	Effect measures	Specify effect measures for each outcome.	Methods, Data Extraction and Data Synthesis	Narrative synthesis; numerical data extracted when available; no pooled effect estimates
13a	Synthesis eligibility	Describe processes for deciding which studies were eligible for each synthesis.	Methods, Data Synthesis	Addressed
13b	Data preparation	Describe methods required to prepare data for presentation or synthesis.	Methods, Data Extraction and Data Synthesis	Addressed
13c	Tabulation and visual display	Describe methods used to tabulate or visually display results.	Results, Tables 3 and 4; Figure 1	Addressed
13d	Synthesis methods	Describe synthesis methods and why they were chosen.	Methods, Data Synthesis	Narrative synthesis due to heterogeneity
13e	Heterogeneity exploration	Describe methods used to explore possible causes of heterogeneity.	Methods, Data Synthesis and Discussion	Narrative comparison by intervention type, age group, context, outcome, and study design
13f	Sensitivity analyses	Describe sensitivity analyses conducted.	Not applicable	No meta-analysis was performed
14	Reporting bias assessment	Describe methods used to assess risk of bias due to missing results.	Limitations	Formal reporting bias assessment was not performed because pooled quantitative synthesis was not conducted
15	Certainty assessment	Describe methods used to assess certainty of evidence.	Methods, Certainty of Evidence; Discussion and Limitations	Formal GRADE was not performed; certainty interpreted narratively
16a	Study selection results	Describe results of search and selection process, from records identified to studies included.	Results, Study Selection and Figure 1	Addressed
16b	Excluded studies	Cite studies that appeared eligible but were excluded and explain why.	Results, Study Selection and PRISMA flow diagram	Addressed using grouped exclusion reasons
17	Study characteristics	Cite each included study and present its characteristics.	Results, Study Characteristics and Table 3	Addressed
18	Risk of bias in studies	Present risk of bias assessments for included studies.	Results, Risk of Bias; Table 4; Supplementary File 2	Addressed
19	Results of individual studies	Present results for all outcomes for each study or group.	Results, Synthesis of Key Findings and Table 3	Addressed narratively
20a	Synthesis results, study characteristics	For each synthesis, briefly summarize characteristics and risk of bias among contributing studies.	Results and Discussion by intervention type	Addressed
20b	Synthesis results, statistics	Present results of statistical syntheses if performed.	Not applicable	No meta-analysis was performed
20c	Synthesis results, heterogeneity	Present results of investigations of heterogeneity if conducted.	Data Synthesis and Discussion	Addressed narratively
20d	Synthesis results, sensitivity analyses	Present results of sensitivity analyses if conducted.	Not applicable	No meta-analysis was performed
21	Reporting biases	Present assessments of reporting bias.	Limitations	Formal assessment not performed due to heterogeneity and no pooled synthesis
22	Certainty of evidence	Present assessments of certainty of evidence.	Methods, Discussion, and Limitations	Narrative certainty assessment reported
23a	Discussion, general interpretation	Provide general interpretation in context of other evidence.	Discussion	Addressed
23b	Discussion, limitations of evidence	Discuss limitations of evidence included in the review.	Discussion and Limitations	Addressed
23c	Discussion, limitations of review process	Discuss limitations of review methods.	Limitations	Addressed
23d	Discussion, implications	Discuss implications for practice, policy, and future research.	Discussion and Conclusion	Addressed cautiously
24a	Registration	Provide registration information for the review.	Methods	This review was not prospectively registered
24b	Protocol access	Indicate where protocol can be accessed.	Methods and Limitations	No prospectively registered protocol
24c	Protocol amendments	Describe and explain amendments to protocol.	Not applicable	No registered protocol
25	Support	Describe sources of financial or non-financial support.	Disclosures/Funding statement	Addressed in manuscript declarations
26	Competing interests	Declare competing interests of review authors.	Disclosures	Addressed in manuscript declarations
27	Availability of data and materials	Report availability of data, code, and other materials.	Methods and tables	Extracted data are summarized in manuscript tables and supplementary material
